# Supplementary material for: A New Species Amecephala micra sp. nov. (Hemiptera: Liadopsyllidae) from Mid-Cretaceous Myanmar Amber
Source: Insects. 2025 Mar 13;16(3):302. doi: 10.3390/insects16030302 (PMC11943161; doi:10.3390/insects16030302)
Supplement: Supplementary file 1 [file insects-16-00302-s001.zip › insects-3504428-supplementary.pdf]

中国科学院南京地质古生物研究所
Nanjing Institute of Geology and Palaeontology, Chinese Academy of Sciences

To whom it may concern:

Each amber piece in the collection of the Nanjing Institute of Geology and Palaeontology has an informal field number (not the accession number provided when published and deposited permanently), indicating when the specimen was collected and by whom. Based on the field numbers of studied specimens, we can trace that the holotype of *Amecephala micra* was bought by Prof. Chenyang Cai and Prof. Diying Huang in late 2015 from a Myanma amber dealer whose family has been working in the amber business for many years. The local amber dealer and his workers mined the raw amber material (not cut, shaped or polished) from Noije Bum, approximately 32 km southwest of Tanai Township. It was transported to Myitkyina for further processing such as trimming, shaping and polishing. From there, jewelry-grade specimens (such as rings and pendants) are carried and sold legally in Ruili county in Dehong Prefecture at the border of China and Myanmar. We can confirm that the amber piece was mined in late 2015, long before the local conflict in the mining area, and it is permanently housed in Nanjing Institute of Geology and Palaeontology, Chinese Academy of Sciences (Nanjing, China).

Chenyang Cai

蔡晨阳，研究员
中国科学院南京地质古生物研究所
江苏省南京市北京东路 39 号
电话/微信：15366104350
Chenyang Cai, Professor
Nanjing Institute of Geology and Palaeontology, Chinese Academy of Sciences
39 East Beijing Road, Nanjing 210008, China
T: +86-15366104350
